# Herbicide resistance status impacts the profile of non-anthocyanin polyphenolics and some phytomedical properties of edible cornflower (*Centaurea* *cyanus* L.) flowers

**DOI:** 10.1038/s41598-023-38520-z

**Published:** 2023-07-17

**Authors:** Urszula Gawlik-Dziki, Barbara Wrzesińska-Krupa, Renata Nowak, Wioletta Pietrzak, Joanna Zyprych-Walczak, Aleksandra Obrępalska-Stęplowska

**Affiliations:** 1 Department of Biochemistry and Food Chemistry, University of Life Sciences, 8 Skromna St, 20-704 Lublin, Poland; 2 Department of Molecular Biology and Biotechnology, Institute of Plant Protection – National Research Institute, 20 Wegorka St, 60-318 Poznań, Poland; 3 Department of Pharmaceutical Botany, Medical University of Lublin, Chodźki 1 Str., 20-093 Lublin, Poland; 4 Department of Mathematical and Statistical Methods, Poznan University of Life Sciences, 28 Wojska Polskiego St, 60-637 Poznań, Poland

**Keywords:** Biochemistry, Enzymes, Metabolomics, Plant physiology, Secondary metabolism, Plant sciences, Plant stress responses, Abiotic

## Abstract

To ensure sufficient food supply worldwide, plants are treated with pesticides to provide protection against pathogens and pests. Herbicides are the most commonly utilised pesticides, used to reduce the growth of weeds. However, their long-term use has resulted in the emergence of herbicide-resistant biotypes in many weed species. Cornflower (*Centaurea* *cyanus* L., Asteraceae) is one of these plants, whose biotypes resistant to herbicides from the group of acetolactate synthase (ALS) inhibitors have begun to emerge in recent years. Some plants, although undesirable in crops and considered as weeds, are of great importance in phytomedicine and food production, and characterised by a high content of health-promoting substances, including antioxidants. Our study aimed to investigate how the acquisition of herbicide resistance affects the health-promoting properties of plants on the example of cornflower, as well as how they are affected by herbicide treatment. To this end, we analysed non-anthocyanin polyphenols and antioxidant capacity in flowers of *C.* *cyanus* from herbicide-resistant and susceptible biotypes. Our results indicated significant compositional changes associated with an increase in the content of substances and activities that have health-promoting properties. High antioxidant activity and higher total phenolic and flavonoid compounds as well as reducing power were observed in resistant biotypes. The latter one increased additionally after herbicide treatment which might also suggest their role in the resistance acquisition mechanism. Overall, these results show that the herbicide resistance development, although unfavourable to crop production, may paradoxically have very positive effects for medicinal plants such as cornflower.

## Introduction

Plant-based food production largely relies on agrochemicals, especially pesticides, used to prevent crop losses caused by pests and pathogens. The highest share of applied pesticides is constituted by herbicides (47.5%) that are used in conventional agriculture to limit crop infestation by weeds. The use of these chemicals has resulted not only in their accumulation in water and soil but also in their presence in the food chain^2^. Pesticides affect their target organisms, a range of non-target species, and plant-associated communities, including microbiota. Furthermore, biodiversity losses are observed and natural habitats for numerous non-target species decline. Eventually, herbicide exposures can lead to substantial compositional shifts within target-plant-associated communities altering interspecies interactions^3^. The generalised herbicide usage has resulted in the evolution of herbicide resistance (HR) in many weed species. HR is one of the most visible problems in agriculture. Two types of mechanisms of HR are observed. The target-site resistance (TSR) is mainly caused by mutations in genes that encode targets for respective active substances in herbicides. This results in the decreased affinity of herbicide-active substances for their target enzyme. The second type of mechanism is referred to as non-target-site resistance (NTSR) and is associated with decreased herbicide uptake and increased xenobiotic detoxification rates^4^.

Flowering weeds are important for pollinators; additionally, their strong root systems protect the soil from erosion and bring minerals to the soil surface^5^. The number of many weed species is, however, steadily decreasing.

Some weed species considered undesired in plant production are highly appreciated in phytomedicine and the food industry. Some of them are valuable sources of medicinal substances and components of dyes and cosmetics. The influence of herbicides, and especially HR, on these properties of weeds is scarcely known. It can be expected that the application of toxic substances might have an impact on the levels and activities of numerous enzymes, a phenomenon that can be maintained over time. It was reported that herbicide resistance may involve modifications in gene expression that can be retained long after exposure to stress, maintaining a ‘stress memory’^6^. In some resistant weed species, the enhanced constitutive expression of a wide range of genes involved in detoxification was observed already before herbicide treatment^7^. Thus, we hypothesised that herbicide treatment and, above all, herbicide resistance development are associated with changes in the levels of some health-promoting substances, e.g., phenolics, especially phenolic acids, flavonoids, and their derivatives.

One of the well-recognised weed species widely used in the phytomedicine and food industry is the cornflower (*Centaurea* *cyanus* L.) from the Asteraceae family. This overwintering species is a melliferous plant pollinated by Apidae, which play an important role in its reproduction^8^. It is becoming increasingly rare in many regions of Europe. Its decline is attributed to some extent to the recent decrease in the number of wild pollinators^8^. However, in some countries, e.g., Czechia, Poland, and Germany, an increase in cornflower populations is observed again. In Sweden, it is frequently found on organic farms^9^. For agriculture, the occurrence of cornflowers in crop fields is problematic, but their presence is important for biodiversity and ecological services. Cornflower contains a wide spectrum of phytochemicals, including phenolic compounds. Dried flowers exhibit antipruritic, antitussive, astringent, weakly diuretic, emmenagogue, and ophthalmic properties. Infusion from cornflower flowers has curative and calming action for nervous disorders^10^. Edible cornflower flowers used as garnish and seasoning are popular food ingredients in Europe^11^. *C.* *cyanus* is a flowering weed indigenous in Asia (Iran, Iraq, Turkey and Pakistan) and Europe (e.g. in Albania, Bulgaria, Greece, Italy) and also cultivated and naturalised in many other parts of the world^12^. In Poland *C.* *cyanus* is also often cultivated for bee food, however cornflower flowers are often obtained from natural sites, because its seeds are expensive.

The cornflower is classified as a plant with a low risk of developing HR^13^. A low number of herbicide-resistant cornflower biotypes have been found according to the International Survey of Herbicide-Resistant Weeds^14^. Tests revealed resistance to herbicides belonging to the group of acetolactate synthase (ALS) inhibitors^15^ (Herbicide Resistance Action Committee (HRAC) group 2), among others to tribenuron-methyl, which is a sulfonylurea herbicide. The molecular basis of HR in the cornflower is poorly known. TSR was found to be involved (Pro197 mutation) in one ALS-inhibitor resistant biotype^16^. Other biotypes showed certain genetic variability present also in herbicide-susceptible biotypes. It was thus suggested that HR might be associated with a NTSR mechanism (or combined TSR and NTSR)^17^.

The subject of the research were cornflower flowers (petals), which are herbal raw material (Flos Cyani—cornflower flower)^18^.

In this study, we have addressed the following issues: (1) can the HR affect the pro-health properties of cornflower flowers? (2) Is HR associated with different levels of polyphenols and antioxidant activities important for phytomedicine? (3) Does herbicide treatment affect the aforementioned properties? The results shed some light on the impact of HR spread on the medicinal quality, composition, and nutraceutical activities in cornflower plants.

## Methods

### Chemicals and plant material

Ferrozine (3-(2-pyridyl)-5,6-bis-(4-phenyl-sulfonic acid)-1,2,4-triazine), ABTS (2,2′-azino-bis(3-ethylbenzthiazoline-6-sulphonic acid)), xantine oxidase (E.C. 1.17.3.2), xanthine, soybean lipoxygenase (E.C. 1.13.11), from *Glycine* *max*, type 1_B, Folin–Ciocalteau reagent, and ammonium thiocyanate were purchased from Sigma-Aldrich (Poznan, Poland). All other chemicals were of analytical grade.

Since the cornflower populations resistant to ALS inhibitors were found thus far only in Poland^14^, plants known for being resistant and susceptible were collected from different regions of the country, where the cornflower control difficulties were reported. A fully developed infruitescences of cornflower were harvested, air-dried, and cleaned. Those seeds were subjected to preliminary test, where the resistance status was confirmed. During this test, plants were sprayed with the recommended dose of Lumer 50 WG (active ingredient (ai) tribenuron-methyl 500 g kg^−1^, ADAMA Agriculture B.V., Schaffhausen, Switzerland) (N—the maximal recommended dose of the herbicide (30 g ha^−1^, i.e. 15 g ha^−1^ of ai)). The control was evaluated visually using a scale from 0 (no effect) to 100% (complete weed destruction) three weeks after application. The populations that passed the preliminary test were then subjected to the detailed test, where chosen tribenuron-methyl susceptible (S) and resistant (R) cornflower biotypes were analyzed (Table 1). Their susceptibility to the herbicide was assessed by the determination of ED_50_ (effective dose of ai causing a 50% reduction in plant biomass). To this aim, the seeds were sown in pots placed under controlled conditions in the greenhouse. Plants at the 12–13 growth stage (according to BBCH scale) were treated with Lumer 50 WG (ai tribenuron-methyl 500 g kg^−1^, ADAMA Agriculture B.V.) at doses: for resistant populations: 0N, 0,5N, 1N, 2N, 4N, 8N, 16N; for susceptible populations: 0N, 1/16N, 1/8N, 1/4N, 1/2N, 1N, 2N, 4N; where N—the maximal recommended dose of the herbicide (30 g ha^−1^, i.e. 15 g ha^−1^ of ai).

**Table 1:** Biotypes used in this study.

Biotype name	Voivodeship	Location	Coordinates	ED50 [ai g h^−1^]
S02	Subcarpathian	Mymoń	49.572598 N, 21.933112 E	< 15
S58	Warmian-Masurian	Bogaczewo	54.10363889 N, 19.57452778 E	
S73	Lubusz	Lubinicko	52.39461111 N, 15.96386111 E	
S83	Opole	Czarnolas	50.58 N, 17.68583333 E	
R80	Warmian-Masurian	Tuławki	53.89806 N, 20.57361 E	> 480
R93	Świętokrzyskie	Sorbice drugie	51.00081 N, 20.14469 E	> 120
R98	Subcarpathian	Sieklówka	49.731767 N, 21.408111 E	> 480

To perform the main experiment, 4 susceptible (S) (S02, S58, S73, and S83) and 3 resistant (R) (R80, R93, and R98) biotypes were sown and placed in controlled conditions in the greenhouse chamber with 16 h light/8 h dark in a temperature of 20 (± 2) °C and humidity of 60%. Fifteen plants per one biotype (5 pots—biological replicates, with 3 plants each) were sown. Plants at the BBCH 12–13 growth stage, 14 days after sowing, were treated with the maximal recommended dose of Lumer 50 WG (30 g ha^−1^). Two days after the treatment, a leaf from one randomly chosen plant from each biotype was harvested for ALS gene sequence analysis. Thereafter, flowers with petals opened with an approximately 90° angle between the petal and stem were cut and stored in the freezer for further analyses. Three flowers per one biological replicate (one flower from each plant) were harvested. Plant material was collected according to the relevant institutional, national, and international guidelines and legislation (permission was obtained from the land owners).

### DNA isolation and ALS sequence analysis

One leaf from the plant from each biotype was ground in a mortar using liquid nitrogen. Genomic DNA was isolated using NucleoSpin Plant II, Mini kit for DNA from plants (Mecherey-Nagel, Düren, Germany) as described^19^. To amplify the cornflower *ALS* sequence, two pairs of primers CcyALSfr and CcyALS12 were used^17^ (Table 2).

**Table 2:** Primers designed to amplify *Centaurea* *cyanus* *ALS* sequence.

Primer name	Primer sequence (5′–3′)	Amplicon length [bp]	Amino acid sequence range* [aa]
CcyALS12	1: TGTTGAGATTACGGGGATTCCGG	991	321–653
	2: CCAGCCGGGATCATGGGCAA		
CcyALSfr	F: CGTKCTBGTRGAAGCCYTSGA	1654–1663	109–663
	R: TCAATATTKYGTTCTKCCATCDCC		

PCR was carried out in a 50-µL reaction mixture containing 1X Q5 Reaction Buffer (NEB, Ipswich, MA, USA), 200 µM dNTPs, 0.5 µM forward and reverse primers (Table S2), 200 ng of genomic DNA, and 1 U of Q5 High-Fidelity DNA Polymerase (NEB). PCR was done in a Mastercycler nexus (Eppendorf, Hamburg, Germany) with an initial denaturation at 98 °C for 30 s, followed by 35 cycles of amplification: 10 s at 98 °C, 30 s at a 63 °C, and 30 s or 1 min at 72 °C, with a final step of 2 min at 72 °C. The reaction products were separated with 1% gel electrophoresis, purified from the gel with Wizard SV Gel and PCR Clean-Up System (Promega, Madison, WI, USA), ligated to pJET1.2 plasmid using CloneJET PCR Cloning Kit (Thermo Fisher Scientific, Waltham, MA, USA), and cloned into DH10B *Escherichia* *coli* competent cells. The plasmids were isolated from *E.* *coli* cells using NucleoSpin Plasmid (Mecherey-Nagel). Three plasmids were sequenced per one plant. The presence of the insert in plasmids was confirmed by the digestion with *BglII*. DNA inserts were sequenced by Genomed (Warsaw, Poland). Sequencing data were analyzed using the BioEdit Sequence Alignment Editor 7.5.5. The obtained sequences were deposited in GenBank.

### Estimation of total phenolics (TPC) and total flavonoids (TFC) and LC–ESI–MS/MS analysis of non-anthocyanin phenolic compounds

Ethanolic extracts (tinctures) from cornflower petals were prepared following Polish Pharmacopoeia IX (https://www.urpl.gov.pl/pl/urz%C4%85d/farmakopea/farmakopea-polska). Two grams of plant material were placed in 50 mL of 70% ethanol and left in darkness for 2 weeks.

Total phenolic content was determined according to the Folin–Ciocalteau method^20^ and expressed as gallic acid equivalents (GAE) per gram of fresh weight (FW). Total flavonoid content was estimated according to Bahorun et al.^21^ and expressed as quercetin (QE) equivalent (mg/g FW).

The phenolic compound profile was analyzed by high-performance liquid chromatography coupled with electrospray ionization mass spectrometry (LC–ESI–MS/MS) using the method proposed by Nowacka et al.^22^ and Pietrzak^23^. An Agilent 1200 Series HPLC system (Agilent Technologies, USA) connected to a 3200 QTRAP Mass spectrometer (AB Sciex, USA) with electrospray ionisation source (ESI) operating in negative-ion mode was used for all analyses. The Analyst 1.5 software (AB Sciex, USA) was used for controlling of separation and data interpretation.

Separation of phenolic and flavonoid compounds was carried out at 25 °C, on a Zorbax SB-C18 column (2.1 × 100 mm, 1.8 mm particle size; Agilent Technologies, USA). The mobile phase consisted of 0.1% aqueous formic acid (solvent A) and acetonitrile with 0.1% formic acid (solvent B). The injection volume was 3 µL and the flow rate was 300 µL/min. The gradient was changed as follows: 0–2 min—20% B; 3–4.5 min—25% B; 5.5–7 min—35% B; 8–12 min—65% B; 14–16 min—80% B. The total run time was 28 min B.

The ESI–MS worked in negative ion mode, and the parameters were as follows: capillary temperature 550 °C, curtain gas at 30 psi, nebulizer gas at 50 psi, source voltage − 4500 V.

Triplicate injections were made for each standard solution and sample. The limits of detection (LOD) and quantification (LOQ) for all analytes were determined at a signal-to-noise ratio of 3:1 and 10:1, respectively. Qualitative identifications of compounds were by comparison of MS/MS spectra and LC retention time with the corresponding standards tested under the same conditions. The calibration curves obtained in multiple reaction monitoring (MRM) mode were used for the quantification of all analytes. The details of the LC–ESI–MS/MS analysis conditions are given in Tables S1, S2. Fragmentation of the chlorogenic acids determined in the samples is given in Fig. S1.

### Antioxidant potential estimation

The ABTS radical scavenging activity was determined according to^24^. The OH• scavenging ability was assessed according to^25^ with some modifications (adopted for microplate reader). The inhibitory activity was determined as EC_50_ [mg FW/mL]- extract concentration providing 50% of activity based on the dose-dependent mode of action.

Reducing power (ferric reducing power, FRAP) was determined according to^26^ and expressed as EC_50_, i.e. the effective concentration at which 0.5 absorbance was achieved.

### Estimation of lipoxygenase-inhibitory (LOXI) and xanthine oxidase–inhibitory (XOI) activity

LOXI activity was determined according to^27^ with modification. The XOI potential was estimated according to^28^, but the method was adapted for the microplate reader. The inhibitory activity was determined as EC_50_ [mg FW/mL], i.e., the concentration of extract providing 50% of activity based on the dose-dependent mode of action.

### Statistical analysis

A two-way ANOVA significance test and Tukey’s pairwise comparisons at a significance level of alpha = 0.05 were used to determine differences in the phenolic content and biological activities between the biotypes. To illustrate the relationship between the variables, the principal component analysis (PCA) was used in the form of PCA biplots. The distance between two points approximates the distance between two observations in the multivariate space (e.g. Euclidean distance). The similarity between the variables was studied by cluster analysis based on the Euclidean distance, and the results were presented in a dendrogram. The complete linkage hierarchical clustering was used. All visualizations were prepared with ggplot2 R package (version 3.4.2). All statistical calculations were performed with the R software, version 3.6.2^29^.

## Results

### ALS sequence analysis

To detect mutations in the *ALS* sequence contributing to HR, the analysis was performed as previously^17^. Sequence variability of the *ALS* in both plants from the R and S biotypes was observed as previously^17^ with no amino acid mutations known for conferring resistance reported in other weed species. Several differences in amino acids were present in the plants of the R and S biotypes. Additionally, mutations at two positions: N404 and V525 were found only in the plants of the R biotypes. Mutations N404R and V525I were found in only one plant of the R80 biotype, and mutation N404K was found in one plant of the R93 biotype. Because no amino acid changes known for conferring resistance to ALS inhibitors were identified, the resistance may be an effect of NTSR or both NTSR and TSR mechanisms. The unique *ALS* nucleotide sequences were deposited in GenBank under accession numbers MW802602-MW802627.

### HR and herbicide treatment of plants causes a shift in flowering time

The treatment with the maximal recommended dose of Lumer 50 WG (30 g ha^−1^) caused over 50% reduction in biomass of plants from the susceptible (S) biotypes (S02, S58, S73, S83). In the resistant (R) biotypes (R80, R93, and R98), the effective dose (ED50) was higher than 120 ai (active ingredient) g h^−1^ in the R93 biotype and 480 ai g h^−1^ in the R80 and R98 biotypes (Table 1).

**Figure 1:**
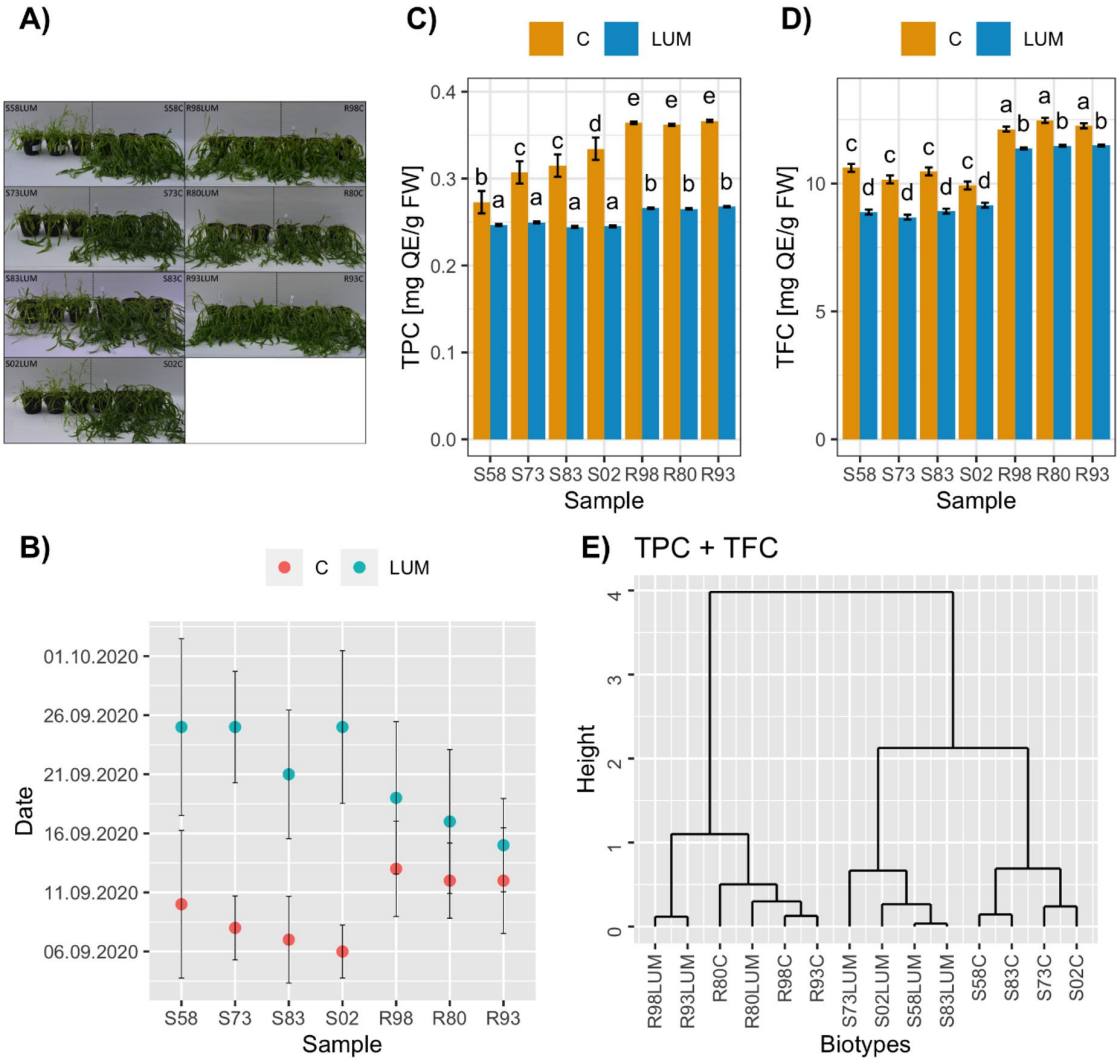
Plant reaction to herbicide treatment (**A**)*, flowering date (**B**)**, total phenolics (TPC) (**C**) and total flavonoids (TFC) (**D**) content, the dendrogram of similarity of the cornflower plants from sensitive (S) and resistant (R) biotypes, treated (LUM) or non-treated (C – control) with herbicide, based on the total phenolics and total flavonoids content (**E**).*Centaurea *cyanus* plants 28 days after treatment with herbicide (Lumer 50 WG, 30 g ha^−1^). Seven biotypes (four sensitive (S) to the left and three resistant (R) to the right) were taken for the analysis. There are 3 pots with non-treated plants (left) and 3 pots with plants treated with Lumer 50 WG (right) for each biotype. **The harvest dates of the first three flowers from each of the five pots from tested biotypes treated or non-treated with the herbicide (with petals opened with an approximately 90° angle between petal and stem).

Herbicide-treated S plants tended to grow less than untreated plants (Fig. 1A). The herbicide treatment led to a delay in the onset of blooming of plants that had survived (on average 48.5 ± 7.0 days after the treatment) compared to the non-treated plants (on average 37.3 ± 4.8 days after the treatment) (Fig. 1B). Plants from the S biotypes started to bloom earlier (on average 35.2 ± 4.3 days after the treatment) than those from the R biotypes (on average 40.1 ± 4.0 days after the treatment). The herbicide treatment resulted in a delay in the onset of blooming of the susceptible plants (on average 51.5 ± 6.4 days after the treatment) in comparison to the resistant ones (on average 44.6 ± 5.9 days after the treatment).

### Plant herbicide resistance status has an impact on the phenolic profile in cornflower flowers

Flowers of plants from the R biotypes had a higher content of total phenolics (TPC) and total flavonoids (TFC) (Fig. 1C,D). This relationship persisted also after the herbicide treatment, although the TPC and TFC levels decreased in both biotypes (Fig. 1C,D). Noticeably, the flowers of the herbicide-treated resistant plants had higher TFC content than the flowers of both the treated and untreated S-plants (Fig. 1D).

The dendrogram shows a clear division between plants from S and R biotypes in terms of TPC and TFC (Fig. 1E). The cluster containing the S biotypes is further divided into two subclusters showing the difference between the herbicide-treated and non-treated plants.

The flowers of both biotypes did not differ in terms of the quality of phenolic acids, but significant differences were related to their quantity. Irrespective of the biotype, 4-hydroxycinnamic, ferulic, and 5-caffeoylquinic acids were the dominant phenolic acids. Significantly higher content of 3-O-caffeoylquinic, 5-caffeoylquinic, vanillic, syringic, and ferulic acids were found in the flowers of the untreated R-plants, while the level of other phenolic acids did not differ significantly between the biotypes (Table 3).

**Table 3:** Average content of phenolic acids, flavonoid aglycones and flavonoid glycosides in flowers of *C.* *cyanus* depending on the plant biotype and herbicide treatment (means of the four sensitive and three resistant biotypes, n = 3. The contents of the tested compounds in the flowers of individual biotypes are presented in Table S3.

Compound	Untreated plants		Treated plants	
	Sensitive	Resistant	Sensitive	Resistant
Phenolic acids [µg/gFM]				
3-O-caffeoylquinic acid*	1.89 ± 0.21^b^	2.52 ± 0.42^c^	1.41 ± 0.35^a^	1.05 ± 0.06^a^
Protocatechuic acid	36.84 ± 0.28^b^	40.45 ± 0.79^b^	19.81 ± 1.28^a^	23.67 ± 2.70^a^
5-caffeoylquinic acid **	60.57 ± 1.4^b^	81.28 ± 2.22^a^	122.07 ± 16.82^c^	98.49 ± 12.26^a^
4-caffeoylquinic acid ***	0.32 ± 0.05^a^	0.47 ± 0.04^ab^	0.81 ± 0.01^c^	0.59 ± 0.04^b^
4-Hydroxybenzoic acid	2.43 ± 0.07^c^	2.03 ± 0.11^c^	0.47 ± 0.07^a^	0.80 ± 0.01^b^
Caffeic acid	16.39 ± 0.27^b^	16.07 ± 0.04^b^	8.81 ± 2.13^a^	7.10 ± 1.21^a^
Vanilic acid	4.42 ± 0.14^b^	5.28 ± 0.42^c^	2.70 ± 0.61^a^	3.61 ± 0.03^b^
Syringic acid	4.43 ± 0.23^b^	11.02 ± 0.10^c^	1.62 ± 0.23^a^	5.52 ± 0.18^b^
4-Hydroxycinnamic acid ****	253.51 ± 12.01^c^	255.08 ± 13.41^c^	88.27 ± 8.79^a^	107.28 ± 3.09^b^
Ferulic acid	115.01 ± 0.20^b^	138.32 ± 2.55^c^	72.55 ± 4.21^a^	72.54 ± 4.55^a^
Flavonoid aglycones [µg/gFM]				
Taxifolin	0.90 ± 0.16^b^	0.82 ± 0.13^b^	0.40 ± 0.05^a^	0.38 ± 0.05^a^
Luteolin	2.08 ± 0.33^c^	1.90 ± 0.25^b^	0.80 ± 0.13^a^	0.60 ± 0.10^a^
Quercetin	3.10 ± 0.44^b^	3.29 ± 0.65^b^	0.92 ± 0.35^a^	0.63 ± 0.39^a^
Apigenin	133.60 ± 6.88^c^	123.60 ± 7.08^a^	55.32 ± 4.59^a^	57.67 ± 3.92^a^
Kempferol	1.99 ± 0.28^c^	1.29 ± 0.47^b^	0.22 ± 0.16^a^	0.27 ± 0.15^a^
Isorhamnetin	1.07 ± 0.61^c^	0.67 ± 0.33^bc^	0.15 ± 0.17^a^	0.18 ± 0.12^ab^
Flavonoid glycosides [µg/gFM]				
Luteolin 3,7′-diglucoside	507.87 ± 16.94^c^	446.45 ± 17.16^b^	267.49 ± 19.42^a^	276.49 ± 37.47^a^
Luteolin-7-O-glucoside	1.23 ± 0.33^ab^	1.34 ± 0.12^b^	0.94 ± 0.39^a^	1.07 ± 0.22^ab^
Quercetin-3-O-glucoside (Isoquercetin)	37.48 ± 4.17^a^	50.54 ± 2.85^b^	37.24 ± 4.78^a^	40.30 ± 2.95^a^
Kaempferol—3-O-glucoside (Astragalin)	59.12 ± 8.71^b^	49.92 ± 9.28^b^	36.24 ± 6.82^a^	34.65 ± 1.53^a^
Isorhamnetin-3-O-glucoside	9.09 ± 3.69^a^	9.75 ± 3.42^a^	6.95 ± 1.72^a^	7.19 ± 1.50^a^
Quercetin 3-O-rhamnoside	0.10 ± 0.00^a^	0.07 ± 0.01^a^	0.97 ± 0.17^c^	0.68 ± 0.17^b^
Apigenin 7-O-glucoside (Apigetrin)	54.12 ± 4.36^b^	70.25 ± 4.50^c^	43.97 ± 3.34^a^	43.83 ± 9.50^a^
Kaempferol-3-O-rhamnoside	5.54 ± 0.68^c^	2.69 ± 0.44^b^	0.74 ± 0.13^a^	0.82 ± 0.23^a^

*Neochlorogenic acid, **chlorogenic acid, *** cryptochlorogenic acid, **** p-coumaric acid.

Different letters in the rows indicate differences between results (*P* < 0.05).

Six flavonoid aglycones: taxifolin, luteolin, quercetin, apigenin, kaempferol, and isorhamnetin were identified in the flowers derived from the untreated plants of both biotypes. Regardless of the biotype, apigenin was the dominant compound. Significant differences in its content were found between the biotypes (average content 133.60 µg/g FM and 123.59 µg/g FM in the flowers of the untreated S and R plants, respectively) (Table 3). It should be noted that flowers from S and R biotypes differed significantly in the content of isorhamnetin (average content 1.07 µg/g FM and 0.67 µg/g FM in the flowers of the S and R plants, respectively) (Table 3).

The flowers of the untreated S and R plants were a rich source of non-anthocyanin flavonoid glycosides, especially luteolin 3′,7′–diglucoside (average content 507.87 µg/g FM and 446.45 µg/g FM for the plants of the S and R biotypes, respectively). The flowers of the untreated R plants contained significantly higher content of quercetin-3-O-glucoside and apigenin 7-O-glucoside. In turn, a significantly higher content of kaempferol-3-O-rhamnoside was found in the flowers of the S-plants (Table 3).

In the herbicide-treated samples (regardless of the biotype), the content of flavonoid aglycones, especially isorhamnetin and kaempferol, decreased most substantially, whereas the smallest decline was exhibited by apigenin and taxifolin (Table 3). The analysis of changes in the content of phenolic acids and flavonoid glycosides showed additionally that the flowers of both S and R herbicide-treated plants were characterised by a drastic increase (but higher in R biotypes) in the quercetin 3-O-rhamnoside content (Table 3). The herbicide caused a significant decrease in the luteolin 3′,7′–diglucoside and kaempferol-3-O-rhamnoside content in the flowers of both S and R plants. The largest decreases were recorded for 4-hydroxycinnamic and ferulic acids. Additionally, the content of 3-O-caffeoylquinic, protocatechuic, vanillic, syringic, 4-hydroxycinnamic, and ferulic acids declined. Interestingly, regardless of the biotype, the content of 5-caffeoylquinic and 4-caffeoylquinic acids increased, especially in the S biotypes (Table 3).

**Figure 2:**
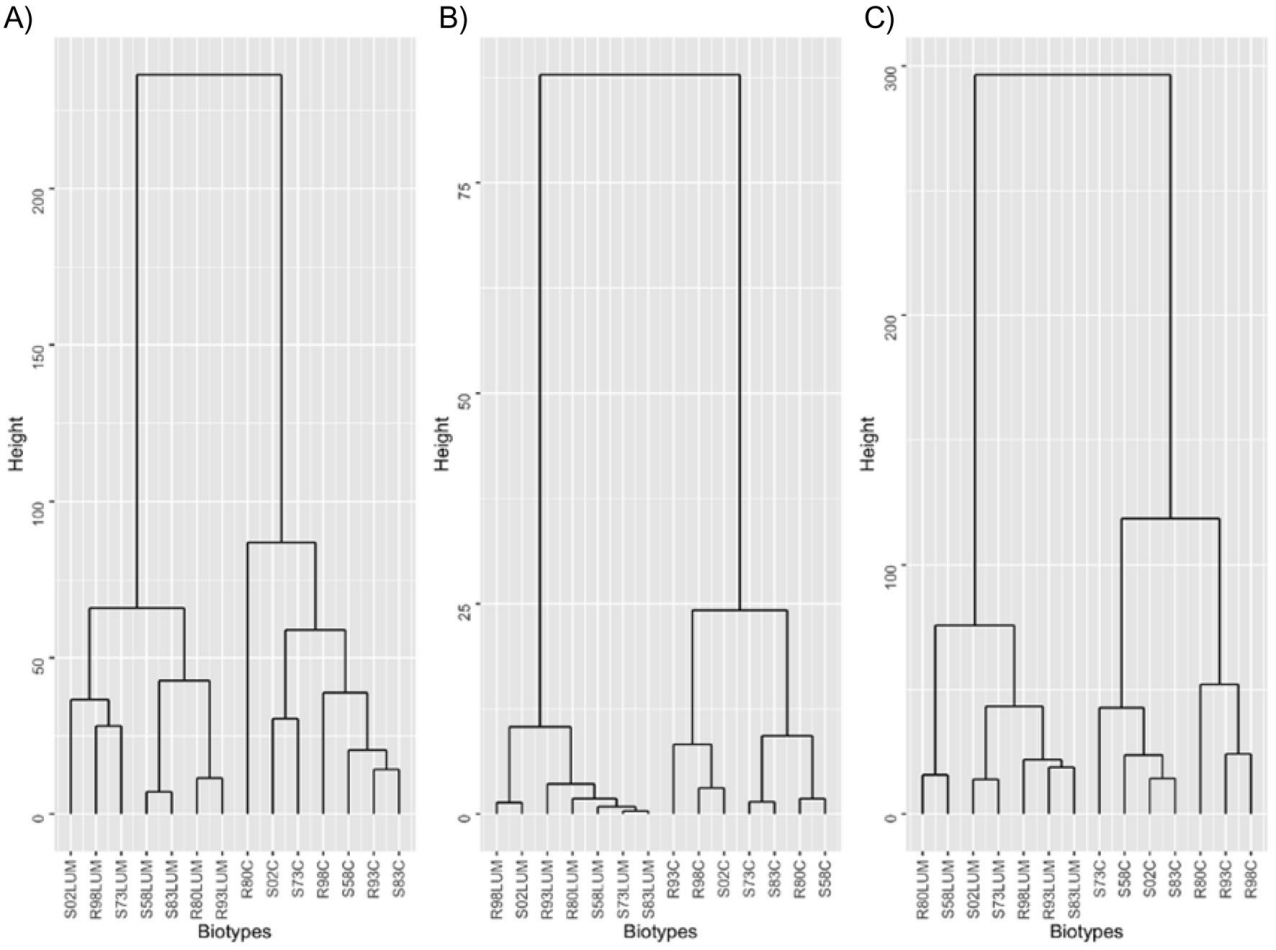
Dendrograms of similarity of the cornflower plants from sensitive (S) and resistant (R) biotypes, treated or non-treated with herbicide based on phenolic acids (**A**), flavonoid aglycones (**B**) and flavonoid glycosides (**C**).

Dendrograms based on the Euclidean distance were drawn to show the content of phenolic acids, flavonoids and flavonoid glycosides (Fig. 2); they demonstrated a division into herbicide-treated and non-treated plants in each case (Fig. 2A–C). However, there was no clear division between the S and R biotypes regarding phenolic acids and flavonoid aglycones. In turn, in the case of flavonoid glycosides, the second cluster of the non-treated plants was also divided into two subclusters containing plants from the S and R biotypes (Fig. 2C).

### Flowers of resistant plants have higher antioxidant potential but the herbicide treatment decreases their antiradical activity

**Figure 3:**
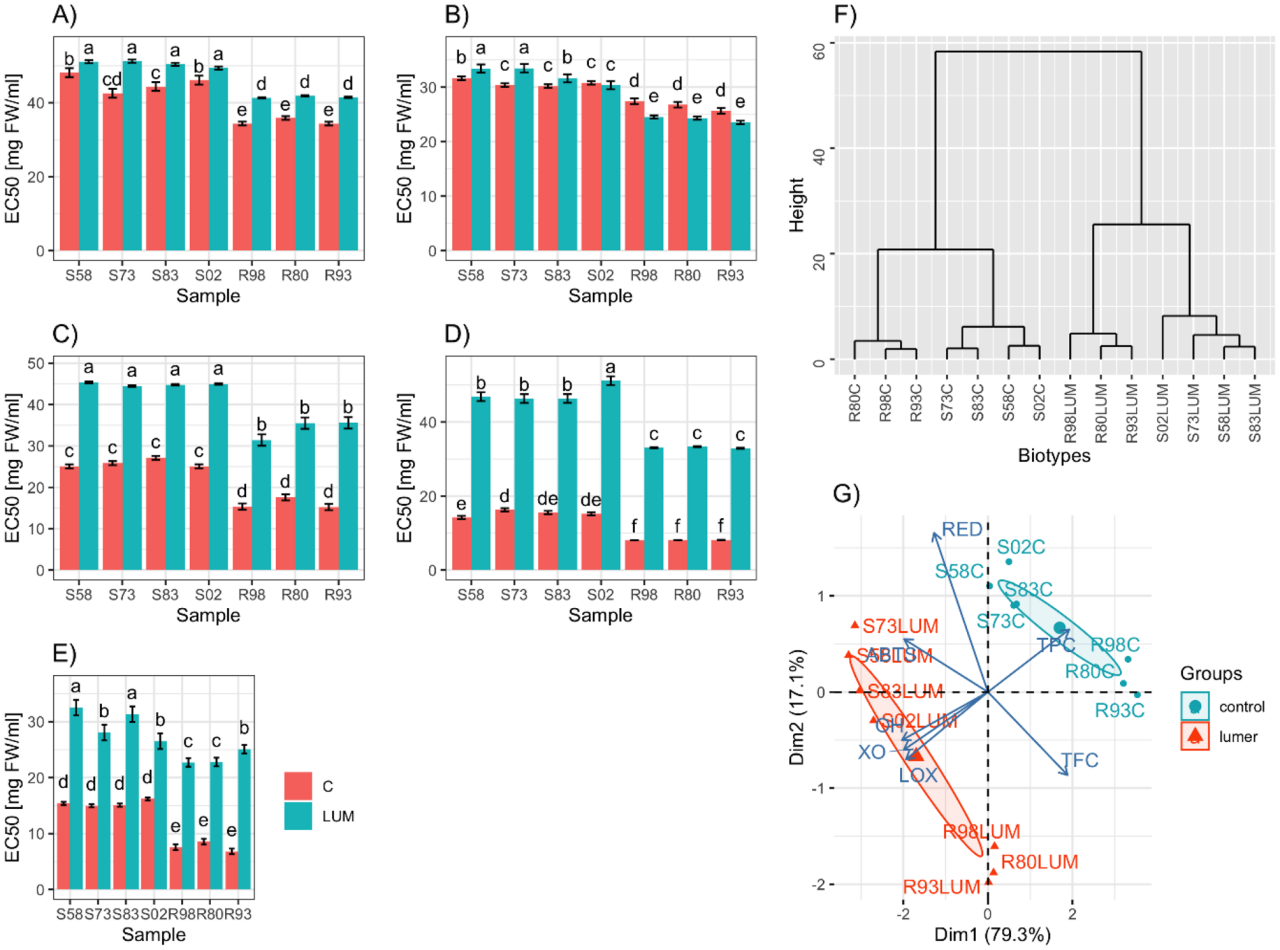
Comparison of antioxidant activity between tinctures from *Centaurea* *cyanus* flowers from sensitive (S) and resistant (R) biotypes, treated with herbicide (LUM) and non-treated (C – control). (**A**) ability to scavenge ABTS free radicals, (**B**) reducing power (RED), (**C**) ability to scavenge hydroxyl free radicals (OH), (**D**) ability to inhibit lipoxygenase activity (LOX), (**E**) ability to inhibit xanthine oxidase (XO) activity. The dendrogram of similarity of the plants from sensitive (S) and resistant (R) biotypes, herbicide (LUM)-treated and non-treated, based on the antioxidant activity (**F**). PCA biplot shows the distribution of the plants from S and R biotypes, treated and non-treated with herbicide (LUM) in relation to the antioxidant activity (**G**).

The flowers of the R-plants had higher antiradical activity (ABTS method) than those of the S-plants (average EC_50_ values = 34.86 and 45.28 mg FW/mL, respectively). Irrespective of the biotype, the herbicide treatment decreased the antiradical activity by about 11% in the S biotypes and by about 19% in the R biotypes. Even after the herbicide treatment, the flowers of the R-plants had higher antiradical activity than those of the susceptible plants (Fig. 3A). The analysis of the reducing power, often referred to as a measure of total antioxidant activity, showed that in the R-plant flowers it was significantly higher. The treatment in these flowers increased the reducing power. In turn, in the case of the S-plant flowers, it resulted in a significant decrease in reducing power in most cases (Fig. 3B).

Regarding the ability to neutralise hydroxyl radicals, significantly higher activity was found in the flowers of the R-plants (average EC_50_ values = 16.06 mg FW/mL and 25.78 for the R and S biotypes, respectively). Moreover, irrespective of the treatment, the flowers of the R-plants showed higher antiradical activity than treated S-plants. In the herbicide-treated plants, the OH∙ radical scavenging activity decreased significantly (about 74% and 112% for the S and R biotypes, respectively) (Fig. 3C).

The flowers of the R- and S-plants showed the ability to inhibit LOX, which was significantly higher in the R-plant flowers (average EC_50_ values = 8.09 and 15.31 mg FW/mL, for the R and S biotypes, respectively). In the flowers of the herbicide-treated plants, the ability decreased markedly; however, this trend was particularly evident in the sensitive biotypes. Regardless of the herbicide use, the flowers of the R-plants had a higher LOX-inhibitory potential than herbicide-treated S-plants (Fig. 3D). A similar tendency was also observed in the inhibition of XO. Namely, the untreated samples were more active (average EC_50_ values = 7.64 and 15.41 mg FW/mL, for the R and S biotypes, respectively). The herbicide treatment caused a drastic decrease in this activity irrespective of the biotype (Fig. 3E).

Euclidean distances based on the antioxidant activity were calculated using the hierarchical clustering complete method, showing two main clusters in the dendrogram: one grouping the herbicide-treated plants and the other comprising the non-treated plants (Fig. 3F). In each of these clusters, biotypes are also grouped according to their S or R biotype. The same division can be seen on the biplot (Fig. 3G) representing the distribution of the herbicide-treated or non-treated plants from the S and R biotypes in relation to their antioxidant activities. The sum of the main PCs indicated that the percentage of information from the original data contained on the biplot is 96.4%. The PCA analysis distinguished four distinct groups (S-treated, R-treated, S-untreated, R-untreated). The angle between the vectors shows that, in the case of OH, XO, and LOX, the correlation of these variables is positive and the most significant in comparison with the RED and ABTS activities, while RED and the group of OH, XO, and LOX are not correlated.

## Discussion

HR is a multifaceted mechanism^30^ involving the synthesis and accumulation of certain specific chemical compounds, many of which have important biological properties crucial for plant protection against herbicides and for human wellbeing. Here, we addressed some aspects related to the latter issue. We asked whether the HR development and spread are associated with the levels of non-anthocyanin polyphenols and antioxidant activities and thus affect the pro-health properties of the cornflower, which is important for phytomedicine and the food industry. Our results provide the first evidence that HR development, although unfavourable to crop production, may have positive effects on medicinal plants by increasing the content of bioactive substances. The cornflower is becoming increasingly rare in many countries in Europe, with ALS inhibitor-resistant biotypes only listed in Poland^14^. So far, only some of the biochemical and physiological aspects of resistant cornflower biotypes exposed to tribenuron-methyl have been investigated^31^. There is no information on the differences in the qualitative and quantitative profile of non-anthocyanin phenolics between the flowers of R and S biotype plants. Our results indicated that the flowers of the R-plants had significantly higher TPC and TFC levels (Fig. 1C,D). A similar phenomenon was observed in grasses, where multiple HR is related to changes in secondary metabolism, particularly the accumulation of flavonoids^32^. The antipathogenic potential of flavonoids can be non-specific and result, in part, from their antioxidative properties^33^. Six flavonoid aglycones and eight flavonoid glycosides were identified in the flowers of the R and S cornflower biotypes (Table 1). Regardless of the biotype, apigenin was the dominant flavonoid aglycone. In turn, luteolin 3’,7’-diglucoside, apigenin 7-O-glucoside and quercetin-3-O-glucoside were the dominant flavonoid glycosides. The presence of these compounds in the cornflower has been confirmed previously^11,34,35^. The content of apigenin 7-O-glucoside and quercetin-3-O-glucoside was slightly higher in the flowers of the R-plants (Table 3). Phenolic acids also play important roles in plant physiology^35^. 4-hydroxycinnamic, ferulic, and 5-caffeoylquinic acids were the dominant phenolic acids identified in the flowers of both biotypes. This is in line with previous reports^11,34^. Importantly, significantly higher content of 3-O-caffeoylquinic, 5-caffeoylquinic, 4-caffeoylquinic, and ferulic acids were found in the flowers of the non-treated R-plants, while the level of other phenolic acids did not differ significantly between the S and R biotypes (Table 3).

It should be emphasized that the level of chlorogenic acid drastically increased after treatment of sensitive plants (S biotype) with the herbicide. An increase in its concentration was also observed in the case of R biotypes, but it was not statistically significant (Table 3). This may indicate the contribution of chlorogenic acids to HR. Chlorogenic acid has been known as a compound that determines resistance to pests and fungi due to its antioxidative properties^34^. Frequently, an increase of ROS is followed by an increase in antioxidants^36^. This was the tendency observed, for instance, by Liu et al. (2019) who used nicosulfuron (ALS inhibitor) in maize (*Zea* *mays* L.)^37^. Decrease of other phenolic acid levels, especially ferulic acid, may be explained by cell wall polymers synthesis^36,38^.

It is difficult to clearly define which specific phenolic compounds play a key role in HR because the differences in their profile are mostly subtle. It seems that HR results from mutual interactions of active compounds responsible for the multidirectional antioxidant activity of these complex systems. Fortunately, all these phenolics have a pro-health effect on humans.

The human organism has a natural antioxidant system that counteracts the effects of oxidants. Antioxidant protection can be enhanced by the intake of exogenous antioxidants. There is a growing consensus that a combination of antioxidants, rather than single entities, may be more effective over a long period of time^39^. The positive effect of plant antioxidants on human health is well documented; phenolic compounds were reported to exert antioxidant, anticancer, antidiabetes, cardiovascular, and anti-inflammatory effects and protective activity in neurodegenerative disorders^40^.

The multidirectional antioxidant potential of the cornflower has been confirmed in our research. A significantly higher antioxidant capacity was exhibited by the flowers of the R-plants. This is especially evident in the ability to neutralise OH∙ radicals physiologically occurring in the human organism, the ability to inhibit XO, i.e. the major human endogenous source of superoxide radicals, and the ability to inhibit the LOX-enzyme (associated with inflammation) (Fig. 3)^34^. The anti-inflammatory properties of cornflower flowers were confirmed by pharmacological experiments^10^. Cornflower extracts inhibited cyclooxygenase 2 (COX-2) and 5-lipoxygenase (5-LOX) and had anti-inflammatory activities mainly due to the presence of N-feruloylserotonin (able to inhibit 5-LOX and COX)^41^, whereas apigenin was the most effective XO inhibitor identified in *C.* *virgata* Lam^42^.

Another aim of the study was to check whether the herbicide treatment changed the non-anthocyanin phenolic profile and health-promoting properties since this might provide some additional cues regarding HR development. Our analyses revealed that TPC and TFC significantly decreased in the flowers of the herbicide-treated plants of both biotypes, but remained at a much higher level in the flowers of the R-plants (Fig. 1C,D). A dramatic drop in the content of flavonoids was observed in wheat after pyroxsulam treatment^43^. It was suggested that the treated wheat plants underwent the induction of stress and oxidant production which is negatively affecting the antioxidant levels. We hypothesise, the impairment of primary metabolism as a result of herbicide treatment results in reduced synthesis of secondary metabolites. Significant changes in the phenolic profile were observed in sulfonylurea-treated sunflowers, where the herbicide exposure induced a strong accumulation of p-coumaric, caffeic, ferulic, and synapic acids^44^.

The herbicide treatment caused a decrease in the content of flavonoid aglycones, especially quercetin and apigenin in the flowers of both R- and S-plants; however, this trend was particularly evident for the R biotypes (Table 3). Probably, quercetin is used for the synthesis of quercetin-3-O-rhamnoside, as evidenced by a significant increase in the content of this compound in the herbicide-treated plants, especially in the treated R-plants (Fig. S1). As reported by Xiao^46^, O-glycosylation reduces the antioxidant activity of flavonoids; however, O-glycosylation can enhance certain types of biological benefits, including antistress activity. This hypothesis is confirmed by the herbicide-induced decrease in antiradical activity observed in our research (Fig. 3A,C). Feeding *Arabidopsis*, tobacco, and duckweed with quercetin suppressed the toxic effects of another herbicide – paraquat (not ALS inhibitor), indicating that this flavonol can be used as an effective protectant against the harmful effects of reactive oxygen species (ROS) on plants growth^4^. The ability of plants to increase the capacity to deal with ROS has been proposed as a mechanism of paraquat resistance^4^. An apparently paradoxical significant decline in antiradical activity was observed in the herbicide-treated plants in this study (Fig. 3A,C). However, the research material constituted flowers collected a long time after the herbicide treatment. It seems that the increase in the antiradical activity described previously is a short-term response to the herbicide action and this property may not be preserved longer.

Noticeably, only in the case of the flowers of the R-plants, a significant increase in reducing power was found after the herbicide treatment (Fig. 3B). It persisted over time after the herbicide exposure, which might suggest its important role in the metabolic NTSR mechanisms of HR.

The herbicide treatment decreased the ability to inhibit LOX and XO. These changes can be explained by the differences in the profile of the active compounds; for example, a decrease in apigenin content may lead to a decrease in the ability to inhibit XO activity^42^.

To the best of our knowledge, the available literature provides no studies on the health-promoting properties, the profile of non-anthocyanin flavonoids and phenolic acids, and the herbicide resistance of edible cornflower parts. The results obtained here form the basis for asking further questions. Since resistant biotypes of cornflowers seem to be valuable sources of healthy compounds, but taking into account that they are herbicide-resistant weeds, can their large-scale cultivation and production be viable? What are the fitness costs on the part of the plant associated with the development of HR?

We have provided data that can be a basis for further phytomedical and ecological research. Recent reports^47^ indicate that climate change might be associated with the earlier onset of a flower blooming. Here, we report that the HR development might in turn lead to a delay in flowering time. The differences in flowering time of the herbicide-resistant and suscepbile plants were also reported previously, with resistant plants blooming earlier^48^, or later^49^ than susceptible ones. Such delay in flowering time may be one of the fitness costs of herbicide-resistant cornflower biotypes. Delayed flowering could potentially affect seed production and interactions with other ecosystem components. Since the flowering time differs between herbicide-resistant and susceptible plants, herbicide exposures may affect flower resources for pollinating insects. Thus, the ultimate effect of this phenomenon would require more extensive research. This finding, together with the described changes in the biochemical composition and activities, shows the far-reaching consequences of human activity on the ecosystem since herbicide-induced phenotype changes can be important for further interactions with other non-target organisms.

## Conclusion

The health-promoting properties of the cornflower were significantly higher in flowers of the resistant biotypes, and although the herbicide treatment caused their decrease, they remained at a higher level than in the treated sensitive plants. It can therefore be assumed that the flowers of the non-treated resistant biotypes are a valuable source of pro-health compounds, better than those of the sensitive biotypes. The high antioxidant capacity, especially reducing power observed in the resistant biotypes and increasing in the herbicide-treated plants, provides further insight into HR mechanisms.

## Supplementary Information

Supplementary Information.

## Data Availability

The dataset generated during the current study concerns ALS nucleotide sequences which were deposited in GenBank (NCBI) under accession numbers MW802602-MW802627.
