# Supplementary material for: Herbicide resistance status impacts the profile of non-anthocyanin polyphenolics and some phytomedical properties of edible cornflower (Centaurea cyanus L.) flowers
Source: Sci Rep. 2023 Jul 17;13:11538. doi: 10.1038/s41598-023-38520-z (PMC10352273; doi:10.1038/s41598-023-38520-z)
Supplement: Supplementary file 1 — Supplementary Information. [file 41598_2023_38520_MOESM1_ESM.docx]

Supplementary data

Figure S1. Fragmentation concerns the chlorogenic acids determined in the samples.


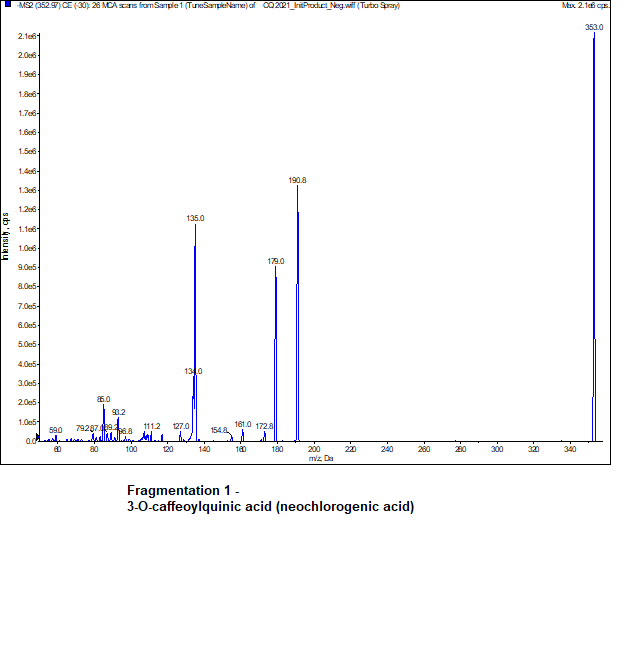

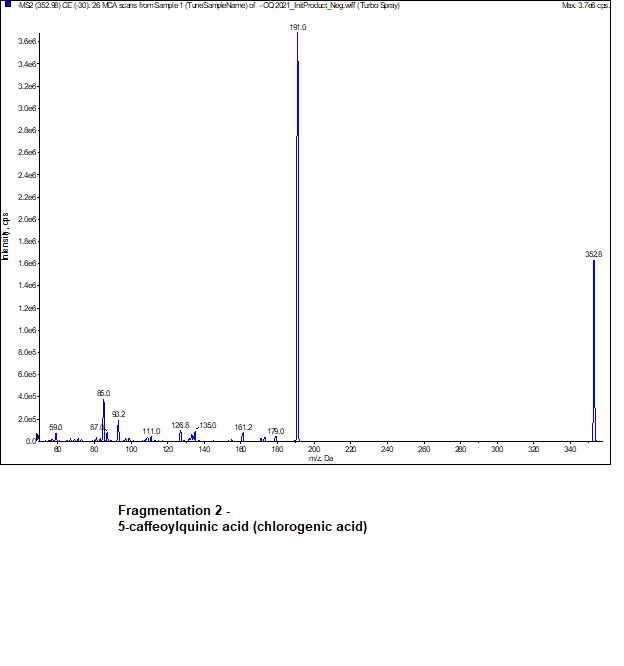

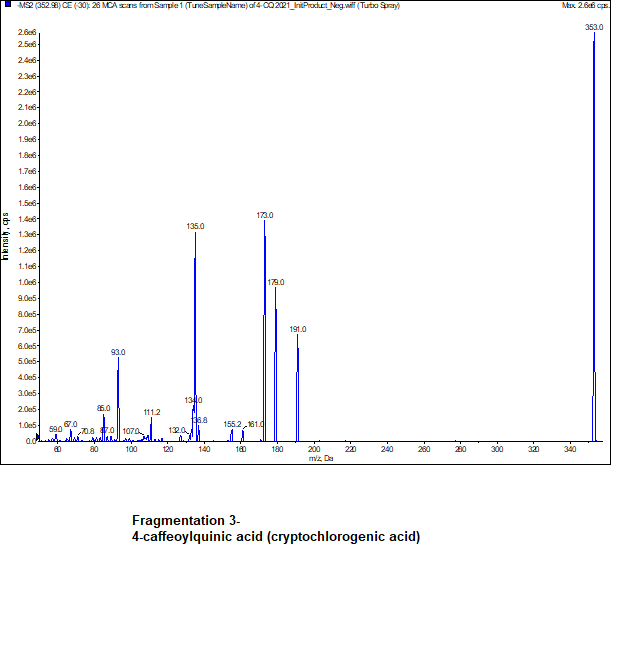


Table S1. LC-ESI-MS/MS analytical results of phenolic acids investigated in samples. Compounds confirmed by comparison with authentic standards.

| **Compound** | **Retention time [min]** | **Q1/Q3**  **[m/z]** | **DP**  **[V]** | | **EP**  **[V]** | **CEP**  **[V]** | **CE**  **[eV]** | **CXP**  **[V]** |
| --- | --- | --- | --- | --- | --- | --- | --- | --- |
| **Phenolic acids** | | | | | | | | |
| Gallic acid | 5.16 | 168.7/78.9  168.7/124.9 | | -35  -35 | -3  -3 | -12  -12 | -36  -14 | 0  0 |
| 3-O-caffeoylquinic acid (neochlorogenic acid) | 6.9 | 353/191  353/178.9 | | -25  -25 | -10  -10 | -25  -25 | -30  -30 | -3  -3 |
| Protocatechuic acid | 8.42 | 152.9/80.9  152.9/107.8 | | -55  -55 | -1  -1 | -10  -10 | -26  -38 | 0  0 |
| 5-caffeoylquinic acid (chlorogenic acid) | 9.30  10.42 | 352.9/190.8  352.9/84.9 | | -35  -35 | -4.5  -4.5 | -16  -16 | -24  -60 | -2  0 |
| 4-caffeoylquinic acid (cryptochlorogenic acid) | 9.4 | 353/173  353/135 | | -25  -25 | -10  -10 | -25  -25 | -30  -30 | -3  -3 |
| 4-Hydroxybenzoic acid | 10.84 | 136.8/92.9 | | -30 | -7 | -10 | -18 | 0 |
| Caffeic acid | 11.38 | 178.7/88.9  178.7/134.9 | | -30  -30 | -6.5  -6.5 | -12  -12 | -46  -16 | 0  0 |
| Vanilic acid | 11.41 | 166.8/107.9  166.8/123 | | -35  -35 | -4  -4 | -12  -12 | -18  -12 | 0  0 |
| Syringic acid | 11.42 | 196.9/122.8  196.9/181.9 | | -30  -30 | -9  -9 | -12  -12 | -24  -12 | 0  -2 |
| 4-Hydroxycinnamic acid (*p*-coumaric acid) | 14.10 | 162.7/119  162.7/93 | | -30  -30 | -8  -8 | -12  -12 | -14  -44 | 0  0 |
| Ferulic acid | 14.80  15.22 | 192.8/133.9  192.8/177.9 | | -25  -25 | -11.5  -11.5 | -14  -14 | -16  -12 | 0  -2 |
| Rosmarinic acid | 15.91 | 358.7/160.8  358.7/196.8 | | -50  -50 | -5  -5 | -26  -26 | -20  -22 | -2  -2 |
| 2-Hydroxycinnamic acid (*o*-coumaric acid) | 16.80 | 162.7/119  162.7/93 | | -25  -25 | -5  -5 | -10  -10 | -14  -46 | 0  0 |
| Salicylic acid | 17.91 | 136.8/93  136.8/75 | | -35  -35 | -4  -4 | -10  -10 | -16  -48 | -2  0 |
| **Flavonoid aglycones** | | | | | | | | |
| Taxifolin | 15.15 | 302.7/124.9  302.7/284.8 | | -45  -45 | -3.5  -3.5 | -18  -18 | -26  -14 | 0  -4 |
| Luteolin | 17.82 | 284.7/132.9  284.7/150.9 | | -75  -75 | -9  -9 | -18  -18 | -38  -26 | 0  0 |
| Eriodictiol | 17.89 | 286.7/134.9  286.7/150.9 | | -45  -45 | -6  -6 | -12  -12 | -32  -18 | 0  -2 |
| Quercetin | 17.94 | 300.7/150.9  300.7/178.8 | | -60  -60 | -2.5  -2.5 | -12  -12 | -26  -20 | 0  -2 |
| Apigenin | 18.64 | 268.8/117  268.8/106.8 | | -70  -70 | -9.5  -9.5 | -12  -12 | -44  -34 | 0  0 |
| Kaempferol | 18.85 | 284.7/116.8  284.7/93 | | -70  -70 | -5  -5 | -12  -12 | -46  -52 | 0  0 |
| Isorhamnetin | 18.99 | 314.7/299.7  314.7/150.9 | | -65  -65 | -2.5  -2.5 | -26  -26 | -20  -30 | -4  0 |
| **Flavonoid glycosides** | | | | | | | | |
| Luteolin 3’,7’-diglucoside | 11.28 | 609.1/285  609.1/447 | | -70  -70 | -7.5  -7.5 | -28  -28 | -50  -32 | -4  -18 |
| Luteolin-7-*O*-glucoside | 12.87 | 446.8/284.8  446.8/132.9 | | -70  -70 | -10.5  -10.5 | -20  -20 | -30  -78 | -4  0 |
| Quercetin -3-*O*-glucoside (Isoquercetin) | 13.00 | 462.7/299.7  462.7/270.7 | | -85  -85 | -1.5  -1.5 | -20  -20 | -30  -44 | -4  -4 |
| Kaempferol – 3-*O*-glucoside (Astragalin) | 14.66 | 446.7/226.8  446.7/254.8 | | -75  -75 | -9  -9 | -20  -20 | -54  -40 | -2  -2 |
| Isorhamnetin-3-*O*-glucoside | 14.76 | 476.8/313.9  476.8/270.9 | | -95  -95 | -10  -10 | -22  -22 | -30  -44 | -4  -4 |
| Quercetin 3-*O*-rhamnoside (Quercitrin) | 14.83 | 446.7/299.7  446.7/270.7 | | -65  -65 | -9  -9 | -18  -18 | -30  -40 | -4  -4 |
| Apigenin 7-*O*-glucoside (Apigetrin, Cosmosiin) | 14.91 | 430.7/267.7  430.7/116.9 | | -70  -70 | -9  -9 | -20  -20 | -38  -84 | -4  0 |
| Kaempferol-3-O-rhamnoside (Azfelin) | 15.9 | 431.1/284.9  431.1/254.9 | | -25  -25 | -10  -10 | -28  -28 | -30  -30 | -3  -3 |

Table S2. Limit of detection (LOD), limit of quantification (LOQ) and calibration curve parameters for phenolic acids.

| **Compound** | **LOD**  **[ng/mL]** | **LOQ**  **[ng/ mL]** | **R^2^** | **Linearity range**  **[ng/ mL]** |
| --- | --- | --- | --- | --- |
| **Phenolic acids** | | | | |
| Gallic acid | 200 | 250 | 0.9985 | 770-38500 |
| 3-O-caffeoylquinic acid (neochlorogenic acid) | 20 | 40 | 0.9996 | 40-10000 |
| Protocatechuic acid | 200 | 250 | 0.9994 | 800-35000 |
| 5-caffeoylquinic acid (chlorogenic acid) | 72 | 180 | 0.9958 | 180-18000 |
| 4-caffeoylquinic acid (cryptochlorogenic acid) | 20 | 40 | 0.9987 | 80-4000 |
| 4-Hydroxybenzoic acid | 200 | 400 | 0.9992 | 400-20000 |
| Caffeic acid | 200 | 400 | 0.9991 | 400-20000 |
| Vanilic acid | 1000 | 1500 | 0.9981 | 1500-20000 |
| Syringic acid | 500 | 732 | 0.9989 | 732-18300 |
| 4-Hydroxycinnamic acid (*p*-coumaric acid) | 100 | 200 | 0.9992 | 400-15000 |
| Ferulic acid | 1000 | 2000 | 0.9985 | 2000-36500 |
| Rosmarinic acid | 500 | 732 | 0.9995 | 732-36600 |
| 2-Hydroxycinnamic acid (*o*-coumaric acid) | 150 | 300 | 0.9984 | 300-10000 |
| Salicylic acid | 500 | 750 | 0.9974 | 1500-15000 |
| **Flavonoid aglycones** | | | | |
| Taxifolin | 10 | 20 | 0.9980 | 40-5000 |
| Luteolin | 10 | 20 | 0.9978 | 30-1500 |
| Eriodictiol | 30 | 60 | 0.9984 | 66-6600 |
| Quercetin | 50 | 100 | 0.9987 | 100-6600 |
| Apigenin | 50 | 100 | 0.9983 | 100-10000 |
| Kaempferol | 300 | 500 | 0.9994 | 500-4470 |
| Isorhamnetin | 150 | 250 | 0.9986 | 250-4470 |
| **Flavonoid glycosides** | | | | |
| Luteolin 3’,7’-diglucoside | 250 | 500 | 0.9988 | 1250-25000 |
| Luteolin-7-*O*-glucoside | 150 | 250 | 0.9983 | 500-25000 |
| Quercetin -3-*O*-glucoside (Isoquercetin) | 50 | 100 | 0.9985 | 1000-50000 |
| Kaempferol – 3-*O*-glucoside (Astragalin) | 100 | 140 | 0.9983 | 2000-35000 |
| Isorhamnetin-3-*O*-glucoside | 100 | 250 | 0.9986 | 2000-25000 |
| Quercetin 3-*O*-rhamnoside (Quercitrin) | 400 | 800 | 0.9995 | 2500-82000 |
| Apigenin 7-*O*-glucoside (Apigetrin, Cosmosiin) | 120 | 300 | 0.9965 | 2000-28800 |
| Kaempferol-3-O-rhamnoside (Azfelin) | 150 | 300 | 0.996 | 300-3000 |

**Table S3.** Content of con-anthocyanin phenolic compounds in flowers of individual *C. cyanus* biotypes untreated and treated with Lumer 50 (n=3)

|  | **Untreated plants** | | | | | | | **Treated plants** | | | | | | |
| --- | --- | --- | --- | --- | --- | --- | --- | --- | --- | --- | --- | --- | --- | --- |
|  | S58 | S73 | S83 | S02 | R98 | R80 | R93 | S58 | S73 | S83 | S02 | R98 | R80 | R93 |
| **Phenolic acids** | | | | | | | | | | | | | | |
| 3-O-caffeoylquinic acid (neochlorogenic acid ) | 1.89 ±0.13^cd^ | 1.70±0.06^e^ | 2.21±0.05^b^ | 1.75±0.04^de^ | 3.08±0.14^a^ | 2.27±0.09^b^ | 2.22±0.03^b^ | 1.14±0.01^fg^ | 1.99±0.16^c^ | 1.27±0.01^f^ | 1.25±0.03^f^ | 1.18±0.06^f^ | 0.98±0.06^g^ | 1.00±0.00^g^ |
| Protocatechuic acid | 31.42±0.28^e^ | 47.53±0.21^a^ | 37.92±0.98^d^ | 30.47±0.53^f^ | 39.92±0.19^c^ | 39.97±0.60^c^ | 41.46±0.20^b^ | 17.30±0.28^k^ | 19.97±0.48^j^ | 19.37±0.12^j^ | 22.59±0.00^i^ | 25.89±0.28^g^ | 20.11±0.20^j^ | 25.00±0.00^h^ |
| 5-caffeoylquinic acid (chlorogenic acid) | 50.99±1.40^h^ | 71.37±2.26^f^ | 65.56±1.18^g^ | 54.35±0.88^h^ | 83.56±2.15^e^ | 98.73±1.4^d^ | 61.55±3.18^g^ | 109.07±0.46^c^ | 148.63±3.38^a^ | 109.73±3.62^c^ | 120.85±1.65^b^ | 123.91±2.24^b^ | 87.82±0.60^e^ | 83.73±2.34^e^ |
| 4-caffeoylquinic acid (cryptochlorogenic acid) | 0.35±0.01^gh^ | 0.25±0.04^i^ | 0.39±0.02^g^ | 0.30±0.02^h^ | 0.53±0.03^e^ | 0.56±0.02^e^ | 0.31±0.02^h^ | 0.63±0.02^d^ | 1.19±0.03^a^ | 0.61±0.02^d^ | 0.82±0.03^b^ | 0.77±0.02^c^ | 0.54±0.01^e^ | 0.46±0.01^f^ |
| 4-Hydroxybenzoic acid | 3.11±0.07^b^ | 1.56±0.11^c^ | 3.44±0.19^a^ | 1.59±0.04^c^ | 3.12±0.08^b^ | 1.53±0.07^c^ | 1.44±0.06^c^ | 0.60±0.02^e^ | 0.44±0.00^d^ | 0.95±0.02^g^ | 0.31±0.00^f^ | 0.53±0.02^e^ | 1.00±0.01^d^ | 0.87±0.02^d^ |
| Caffeic acid | 16.02±0.^27c^ | 14.65±0.04^d^ | 16.29±0.06^c^ | 18.58±0.035^a^ | 16.89±0.18^b^ | 17.10±0.42^b^ | 14.22±0.19^d^ | 6.27±0.17^j^ | 11.13±0.11^e^ | 7.38±0.06^h^ | 10.47±0.06^f^ | 8.61±0.54^g^ | 6.81±0.01^i^ | 5.90±0.01^j^ |
| Vanilic acid | 4.43±0.14^e^ | 5.22±0.02^c^ | 1.62±0.04^k^ | 6.43±0.02^a^ | 5.81±0.10^b^ | 5.06±0.28^cd^ | 4.97±0.08^d^ | 2.52±0.07^i^ | 2.20±0.12^j^ | 3.68±0.11^g^ | 2.38±0.01^ij^ | 3.15±0.04^h^ | 3.97±0.08^f^ | 3.70±0.01^g^ |
| Syringic acid | 6.13±0.23^b^ | 2.81±0.10^f^ | 5.29±0.18^c^ | 3.50±0.16^d^ | 24.21±0.61^a^ | 3.32±0.26^de^ | 5.52±0.14^c^ | 2.00±0.09^g^ | 0.76±0.06^h^ | 0.77±0.08^h^ | 2.94±0.15^ef^ | 5.47±0.03^c^ | 5.36±0.00^h^ | 5.73±0.24b^c^ |
| 4-Hydroxycinnamic acid  (*p*-coumaric acid) | 207.51±2.79^e^ | 284.30±0.41^a^ | 227.78±0.79^f^ | 267.41±1.76^b^ | 241.02±0.59^c^ | 286.72±1.99^a^ | 237.49±1.19^d^ | 78.85±0.74^k^ | 101.54±1.21^i^ | 78.75±1.57^k^ | 93.95±0.51^j^ | 106.23±0.14^h^ | 111.26±0.30^g^ | 104.37±0.21^h^ |
| Ferulic acid | 94.63±0.21^d^ | 112.57±0.55^e^ | 123.96±0.61^c^ | 105.55±0.98^f^ | 115.64±0.04^de^ | 167.54±0.68^a^ | 131.79±0.08^b^ | 58.13±0.24^k^ | 72.59±0.81^i^ | 64.56±0.66^j^ | 94.90±0.25^g^ | 62.66±1.08^j^ | 75.55±0.17^i^ | 79.41±1.48^h^ |
| **Flavonoid aglycones** | | | | | | | | | | | | | | |
| Taxifolin | 0.50±0.03^c^ | 1.11±0.02^a^ | 0.72±0.033^d^ | 0.95±0.04^b^ | 0.73±0.05^d^ | 0.99±0.06^b^ | 0.74±0.05^d^ | 0.34±0.00^f^ | 0.45±0.00^e^ | 0.36±0.03^f^ | 0.43±0.01^e^ | 0.38±0.03^ef^ | 0.44±0.03^e^ | 0.33±0.00^f^ |
| Luteolin | 1.71±0.01^f^ | 2.26±0.07^b^ | 2.48±0.01^a^ | 1.84±0.09^e^ | 1.97±0.05^d^ | 2.13±0.14^c^ | 1.59±0.04^g^ | 0.67±0.02^j^ | 0.84±0.02^i^ | 0.72±0.04^ij^ | 0.98±0.01^h^ | 0.73±0.03^ij^ | 0.54±0.03^k^ | 0.52±0.01^k^ |
| Quercetin | 3.07±0.10^c^ | 2.73±0.10^d^ | 3.78±0.18^b^ | 2.68±0.23^d^ | 4.11±0.18^a^ | 3.06±0.12^c^ | 0.61±0.03^h^ | 0.61±0.03^h^ | 0.89±0.09^fg^ | 0.70±0.03^gh^ | 1.47±0.00^e^ | 0.28±0.01^i^ | 1.14±0.01^f^ | 0.47±0.01^hi^ |
| Apigenin | 138.95±0.41^a^ | 140.28±0.78^a^ | 124.13±1.76^c^ | 122.51±1.76^c^ | 132.20±0.40^b^ | 116.08±1.61^d^ | 138.95±0.41^a^ | 53.26±0.92^fg^ | 52.47±1.56^g^ | 52.73±0.00^g^ | 62.83±1.65^e^ | 62.75±1.54^e^ | 54.25±0.20^fg^ | 56.02±0.43^f^ |
| Kempferol | 1.94±0.06^b^ | 1.73±0.03^c^ | 2.43±0.01^a^ | 0.79±0.05^e^ | 1.86±0.12^b^ | 1.22±0.03^d^ | 1.94±0.06^b^ | 0.31±0.01^g^ | 0.12±0.01^h^ | 0.05±0.00^hi^ | 0.42±0.00^f^ | <LOQ^i^ | 0.41±0.02^fg^ | 0.14±0.02^h^ |
| Isorhamnetin | 0.42±0.00^e^ | 0.77±0.05^c^ | 0.73±0.02^cd^ | 2.08±0.02^a^ | 0.29±0.01^f^ | 1.04±0.03^b^ | 0.68±0.06^d^ | 0.10±0.01^g^ | 0.05±0.00^gh^ | 0.01±0.00^h^ | 0.43±0.01^e^ | <LOQ^i^ | 0.29±0.02^f^ | 0.07±0.01^g^ |
| **Flavonoid glycosides** | | | | | | | | | | | | | | |
| Luteolin 3’,7’-diglucoside | 501.396±5.59^ab^ | 485.47±0.00^b^ | 522.22±3.93^a^ | 522.39±14.07^a^ | 453.04±27.35^c^ | 428.19±1.63^d^ | 458.12±7.95^c^ | 240.86±2.77^hi^ | 273.89±1.21^fg^ | 291.81±2.41^ef^ | 261.66±3.09^gh^ | 299.41±6.99^ef^ | 227.34±3.00^i^ | 302.71±17.04^e^ |
| Luteolin-7-O-glucoside | 0.95±0.08^d^ | 1.75±0.00^a^ | 0.96±0.04^d^ | 1.26±0.08^c^ | 1.49±0.03^b^ | 1.27±0.00^c^ | 0.95±0.08^d^ | 0.47±0.02^g^ | 1.52±0.00^b^ | 0.90±0.02^de^ | 0.87±0.012^e^ | 1.21±0.01^c^ | 0.77±0.01^f^ | 1.22±0.02^c^ |
| Quercetin -3-O-glucoside (Isoquercetin) | 44.31±0.84^c^ | 42.30±2.26^cd^ | 39.86±2.55^de^ | 35.45±0.53^f^ | 48.76±1.76^b^ | 54.24±0.00^a^ | 48.62±0.39^b^ | 31.01±1.38^g^ | 39.16±1.81^e^ | 35.58±1.33^f^ | 43.22±0.93^c^ | 44.17±0.98^c^ | 38.24±0.40^ef^ | 38.48±0.96^e^ |
| Kaempferol – 3-O-glucoside (Astragalin) | 52.86±1.26^c^ | 72.38±2.88^a^ | 51.81±0.59^cd^ | 59.58±3.34^b^ | 40.06±1.95^f^ | 48.45±0.20^de^ | 61.27±0.79^b^ | 34.20±0.00^g^ | 33.62±0.72^g^ | 47.10±2.65^e^ | 30.03±1.24^h^ | 34.78±0.56^g^ | 32.86±0.60^gh^ | 36.30±0.43^g^ |
| Isorhamnetin-3-O-glucoside | 9.33±0.25^d^ | 5.24±0.25^h^ | 14.66±0.19^a^ | 6.02±0.19^efg^ | 13.87±0.04^b^ | 9.36±0.44^cd^ | 9.33±0.25^cd^ | 6.49±0.10^e^ | 5.78±0.24^g^ | 5.76±0.13^g^ | 9.75±0. 04^c^ | 6.43±0.07^ef^ | 9.16±0.29^d^ | 5.97±0.26^fg^ |
| Quercetin 3-O-rhamnoside | 0.10±0.00^f^ | <LOQ^g^ | <LOQ^g^ | <LOQ^g^ | 0.07±0.02^f^ | <LOQ^g^ | 0.10±0.00^f^ | 0.72±0.04^d^ | 1.00±0.04^b^ | 1.19±0.04^a^ | 0.96±0.02^b^ | <LOQ^g^ | 0.84±0.01^c^ | 0.52±0.04^e^ |
| Apigenin 7-O-glucoside (Apigetrin) | 51.51±0.84^f^ | 60.17±0.41^d^ | 55.42±0.19^e^ | 49.38±2.29^g^ | 64.36±0.39^c^ | 72.32±1.19^b^ | 74.06±0.19^a^ | 38.45±0.28^i^ | 46.08±0.24^h^ | 45.73±0.00^h^ | 45.63±0.41^h^ | 56.23±0.14^e^ | 39.87±0.30^i^ | 35.39±0.00^i^ |
| Kaempferol-3-O-rhamnoside | 6.18±0.17^a^ | 5.99±0.16^a^ | 4.53±0.12^c^ | 5.45±0.03^b^ | 2.30±0.10^e^ | 2.51±0.15^e^ | 3.25±0.02^d^ | 0.58±0.01^h^ | 0.73±0.03^gh^ | 0.70±0.03^gh^ | 0.93±0.00^fg^ | 0.68±0.04^h^ | 0.67±0.01^h^ | 1.12±0.00^f^ |

LOQ -limit of quantification. Different letters in the rows indicate differences between results (P < 0.05)
